# Novel insights into iron regulation and requirement in marine medaka *Oryzias melastigma*

**DOI:** 10.1038/srep26615

**Published:** 2016-05-24

**Authors:** Jian Wang, Wen-Xiong Wang

**Affiliations:** 1 Division of Life Science, State Key Laboratory of Marine Pollution, Hong Kong University of Science and Technology (HKUST), Clearwater Bay, Kowloon, Hong Kong

## Abstract

Iron (Fe) is an essential trace element for marine fish. However, our knowledge of Fe requirements at different development stages of marine fish is still limited. Here, we reported the efficient Fe absorption strategies adopted by larval fish under different dietary Fe supplementary levels (i.e., 0–640 mg/kg). Biokinetically, the larval fish controlled their dietary Fe assimilation efficiency (AE, 1.6–18.5%), and enhanced their waterborne Fe uptake (ca. 2.5 fold change of uptake rate constant) once the dietary Fe was deficient (i.e., 27.4 mg Fe/kg feed). Transcriptionally, the expression of hepcidin1 (*hep1*; Fe regulator; i.e., 2.3–15.7 fold change) in larval fish was positively correlated with the Fe supplementary levels. Comparatively, the female adult fish were poor in assimilating the added Fe source (i.e., ferric form) with similar life-sustainable levels of Fe (i.e., 0.046–0.12 μg/g/d assimilated for Fe supplementary levels of 27.4, 162 and 657 mg Fe/kg feed). The overall feeding experiments suggested that dietary net Fe flux sufficient for the normal growth of larval medaka was 0.71–1.75 μg/g/d (i.e., 83.9 mg Fe/kg feed), consistent with the modeled value (i.e., 1.09–2.16 μg/g/d). In female adults, the estimated essential net Fe flux was 0.88–0.90 μg/g/d.

During the development of a single embryo, iron (Fe) is actively involved in various biological processes (e.g., oxygen transfer, DNA synthesis and immune function). Fe requirement in fish is conventionally quantified by an optimal dietary inclusion concentration (i.e., 21–166.7 mg Fe/kg)^1^,^2^,^3^, but there are some inherent disadvantages of this method. Firstly, there is ambiguity of the determined optimal feed Fe concentration. Over a certain supplementary level (typically >30 mg Fe/kg feed), the dose-dependent response was not obvious in most of the feeding experiments^2^. Rigos *et al*.^4^ fed the Gilthead seabream with Fe supplementary feed (i.e., 5–309 mg Fe/kg feed) for 12 weeks, but no significant difference was detected in growth and tissue Fe distribution among different Fe treatments^4^. Similar results were also found in the Fe nutritional studies on Atlantic salmon (*Salmo salar*), yellowtail (*Seriola quinqueradiata*), tilapia (*Oreochromis niloticus×O. aureus*) and carp (*Cyprinus carpio var. Jian*)^5^,^6^,^7^,^8^. Thus, the determined optimal feed Fe concentration was highly affected by the initial designated dietary Fe concentrations. Secondly, factors such as Fe form and diet substance can affect the bioavailability of Fe to fish. For example, ferrous ions could be more efficiently transported across the intestinal barrier than ferric ions in the European flounder (*Platichthys flesus*)^9^. This was further supported by the evidence that diet supplemented with ferrous sulfate was able to meet the Fe requirements of tilapia (*Oreochromis niloticus*×*O. aureus*), whereas diets supplemented with ferric citrate was not^7^. The presence of phytate in the fish feed could decrease the bioavailability of Fe (forming insoluble salt), which can be relieved by the additional supplement of phytase^10^,^11^. Thirdly, previous study on Zn requirement in fish suggested that higher Zn flux was needed in juvenile stage than in post-juvenile stage^12^, and similar stage-dependent Fe requirement may also occur. Concerning these factors, our knowledge of the basic biological Fe requirements in fish is still lacking, not to mention its underlying regulation.

Zhao *et al*. proposed an intestinal Fe absorption pathway of zebrafish, which was analogous to that reported in mammalian systems^13^. Briefly, the Fe absorption process involved firstly with the transformation of trapped ferric Fe to ferrous Fe and then transport in the enterocytes by divalent metal transport 1 (DMT1). The ferrireduction process is generally considered as one of the rate limiting steps controlling the Fe internalization. Fe is then removed from the enterocytes through Fe-regulated transporter (FPN1) and oxidized by hephaestin. The exportation process is controlled by hepcidin (HEP). Once Fe is overloaded, the expression of HEP is up-regulated and the excessive HEP can specifically bind with FPN1, which then triggers the internalization of FPN1. The exported Fe then binds with transferrin (TF) and is further transported to the target cell by recognition of transferrin receptor (TFR). The incorporated Fe can be subsequently used in various biological processes and the extra ones are stored in ferritin. Comprehensive studies on Fe regulation have been conducted in mammals, which was highly dependent on the modulation of dual functional (i.e., Fe regulator and antimicrobial agent) hepcidin (*hep*) gene^14^,^15^. Several homologs of the *hep* gene have been identified in fish^16^. Neighbor-Joining analyses suggested the sub-functionalization of these homologs, which may be related to the diversity of aquatic habitats with varying degrees of pathogen challenge, oxygenation and Fe concentration^16^,^17^. An earlier study demonstrated the dual function of hepcidin in sea bass (*Dicentrarchus labrax*)^18^, but recently the sub-functionalization of these *hep* genes (*hep1* for Fe regulation and *hep2* for antimicrobial) was also discovered^19^. Similar phenomenon was also found in turbot (*Scophthalmus maximus L*.) that both *hep1* and *hep2* were affected by pathogen challenges, while only *hep1* was up-regulated after Fe overloading^20^. Therefore, the *hep* is also actively involved in Fe regulation in fish, but the relative importance is still unknown compared to other Fe absorption related genes (e.g., *dmt1*, *fpn1* and *dcytb;* see Table 1 for full name).

To fill these research gaps, the present study systematically investigated the Fe regulation and requirement in marine medaka. Briefly, the critical Fe requirements at different life stages (i.e., larval and female adult) were identified through whole body Fe content screen. A conventional 2-week feeding experiment was subsequently conducted to investigate their Fe regulation and requirement. Fe regulation was studied at both biokinetic and transcriptional levels at different time intervals over 2-week feeding experiment. Stage-dependent Fe requirement was determined based on these responses and quantified using universal applicable net Fe flux. A modified fractional model developed by Wang and Wang^12^ was also applied to estimate the essential daily net Fe flux in medaka as well as some other fish species at different life stages.

## Results

### Whole life stage Fe content assay

**Figure 1:**
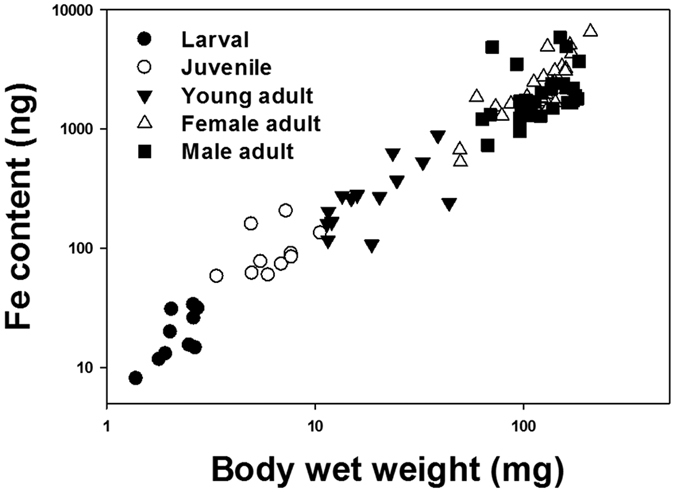
Total body iron content over different developmental stages (n = 95 in total). For the larval and juvenile stages, each point included 10–30 individuals. For young adult and adult, only 1 individual was sampled for each point.

The overall fish body Fe content increased from ca. 6 ng to ca. 6000 ng and the body weight increased from ca. 1 mg to ca. 200 mg throughout the whole developmental stage (Fig. 1). Specifically, four life stages were determined according to the study on a similar specie *Oryzias latipes*^21^. By conducting linear regression of the log transformed body weight and Fe content, slope larger than 1 (increasing Fe concentration) was observed in the larval (b = 1.6416, r^2^ = 0.72) and female adult (b = 1.1749, r^2^ = 0.61) stages. Juvenile and young adult stage showed a slope close to 1, corresponding to a constant body Fe concentration, whereas a poor correlation (r^2^ = 0.16) was observed in male adult stage. Therefore, larval and female adult stages were identified as the critical Fe requirement stages in the present study.

### Growth performance and body metal concentration

No mortality was recorded for the larval fish in all treatments over 2-week exposure. No significant difference (*p* > 0.05, one-way ANOVA) was detected in specific body weight growth rate (SWG; ca. 8.0%/d) (Table 2). Only 335 mg Fe/kg treatment showed a significant (*p* < 0.05, one-way ANOVA) larger specific body length growth rate (SLG; i.e., 2.31%/d) and total consumed feed (TCF; i.e., 6.01 mg) than the rest treatments. However, fish fed 27.4 mg Fe/kg diet had a significantly (*p* < 0.05, one-way ANOVA) higher Fe (i.e., 356 mg/kg) and Cu (i.e., 11.7 mg/L) concentration than the rest treatments. Besides, fish body Zn concentration varied slightly (i.e., 177–206 mg/kg) among different treatments.

The Fe concentration of the female adult fish was assayed in different tissues. Briefly, no significant difference (*p* > 0.05, one-way ANOVA) of Fe concentration in gill (i.e., 60.0–81.9 mg/kg), liver (i.e., 21.9–33.4 mg/kg) and carcass (i.e., 12.7–14.1 mg/kg) were detected among the three different Fe levels (i.e., 27.4, 162 and 657 mg Fe/kg), while only 657 mg Fe/kg treatment had a significantly higher (*p* < 0.05, one-way ANOVA) ovary Fe concentration (i.e., 44.18 mg/kg) than the other two treatments (i.e., 5.81–13.4 mg/kg).

### Biokinetic responses

**Figure 2:**
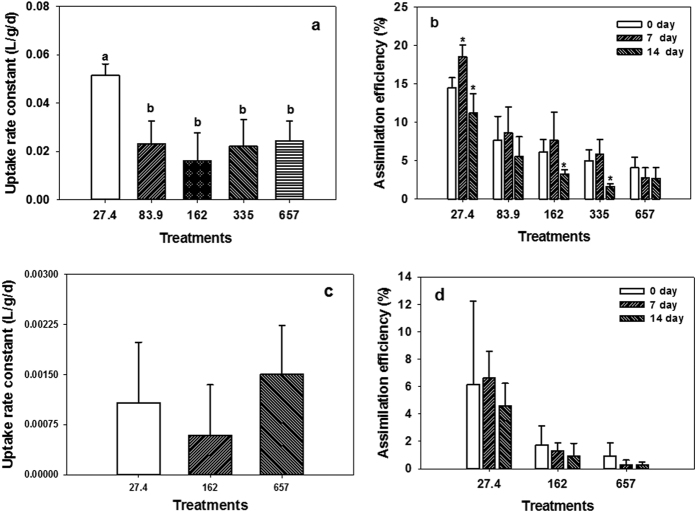
Biokinetic responses of medaka over 2-week of feeding, (**a**) short term uptake rate constant (k_u_) and (**b**) assimilation efficiency (AE) on day 0, 7 and 14 for larval fish; (**c**) k_u_ and (**d**) AE on day 0, 7 and 14 for female adult fish. Values (n = 3) are expressed as Mean ± SD. As for k_u_, bars sharing the same alphabet were statistically not significant (*p* > 0.05, one-way ANOVA). For the same dietary Fe supplementary level, AE significant from day 0 was marked with asterisk (*p* < 0.05, one-way ANOVA).

Biokinetic responses in larval fish were quantified using k_u_ and AE (Fig. 2a,b). After 2-week feeding experiment, fish fed 27.4 mg Fe/kg diet had a more than 1.5 times higher k_u_ than the remaining treatments, while only a slight variation of k_u_ was observed among the 83.9–657 mg Fe/kg treatments. The AE values changed significantly (*p* < 0.05, two-way ANOVA) over 2-week feeding experiment. Specifically, a significant (*p* < 0.05, one-way ANOVA) increase of AE (from 14.5% to 18.5%) on day 7 was observed in fish fed 27.4 mg Fe/kg diet. However, the AE decreased on day 14 in all treatments with significant difference detected (*p* < 0.05, one-way ANOVA) in 27.4, 162 and 335 mg Fe/kg treatments. Besides, the AE decreased significantly (*p* < 0.05, two-way ANOVA) with the increase of Fe supplementary levels.

As for female adult fish, no significant difference (*p* > 0.05, one-way ANOVA) of k_u_ (ca. 0.001 L/g/d) was detected among all the treatments (Fig. 2c). The AE decreased (from 6.2% to 0.94%) with the increase of Fe supplementary levels, but did not change significantly (*p* < 0.05, two-way ANOVA) for each supplementary level over 2-week feeding experiment (Fig. 2d).

### Transcriptional responses

**Figure 3:**
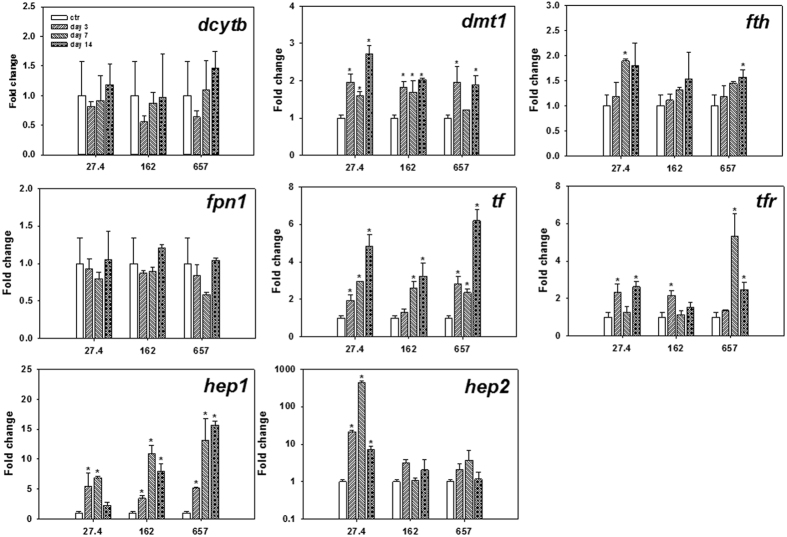
Transcriptional responses of larval medaka over 2-week feeding experiment for different Fe levels (27.4, 162 and 657 mg Fe/kg). Values (n = 3) are expressed as mean fold change ± SD against day 0, and significant differences (*p* < 0.05, one-way ANOVA) from day 0 (ctr) were marked with asterisk.

For larval fish, transcriptional responses were only evaluated at three different Fe supplementary levels (i.e., 27.4, 162 and 657 mg Fe/kg; Fig. 3). The Fe absorption related gene expressions were categorized into two groups. The expression level of the Fe exporter (*fpn1*) and Fe importation facilitator (*dcytb*) did not change significantly (*p* > 0.05, two-way ANOVA) over 2 weeks for all the treatments. Significant (*p* < 0.05, two-way ANOVA) up-regulations were observed for the hepcidin genes (i.e., *hep1*and *hep2*) and genes involved in intracellular Fe transportation activities (i.e., *tf*, *tfr*, *dmt1* and *fth*) over 2 weeks. Briefly, *hep1* expression level increased (from 2.3 to 15.7 fold) in line with the elevation of Fe supplementary levels over 2-week feeding experiment. Besides, *hep1* kept increasing during the first week and arrived at a plateau thereafter for the 162 and 657 mg Fe/kg treatments, while a declined expression level to the initial state (day 0) occurred for the 27.4 mg Fe/kg treatment. Remarkable fold change of *hep2* was observed in the 27.4 mg Fe/kg treatment, with the highest fold change (i.e., 443.7) reached on day 7, while only a slight variation occurred in the 162 and 657 mg Fe/kg treatments. The *dmt1* was significantly (*p* < 0.05, one-way ANOVA) up-regulated in nearly all the treatments (except 162 mg Fe/kg treatment on day 14) over 2-week exposure and stayed at a relatively constant level (i.e., 1.5–2.7 fold). The expression level of *tf* kept increasing (i.e., 1.3–6.2 fold) over 2-week feeding experiment for nearly all the treatments, except a plateau on day 7 for the 657 mg Fe/kg treatment. Expression of *tfr* was up-regulated slightly (i.e., fold change <3.0) in 27.4 and 162 mg Fe/kg treatments, but was strongly up-regulated (i.e., 5.3 fold change) in 657 mg Fe/kg treatment on day 7.

**Figure 4:**
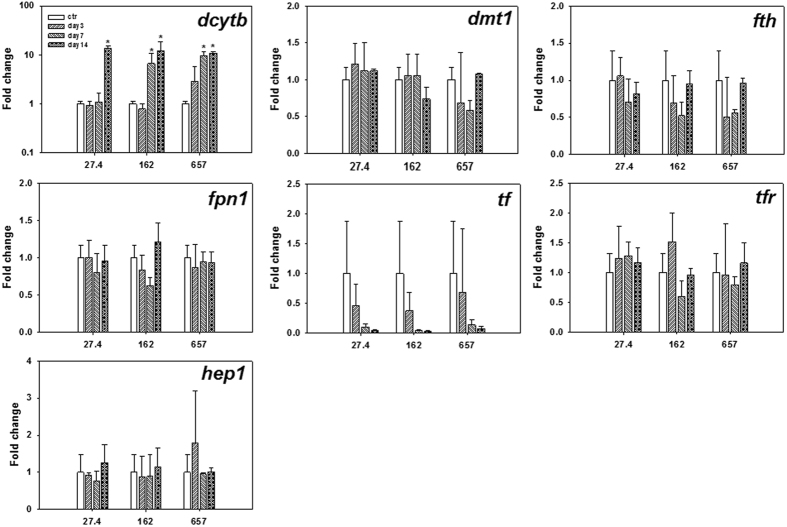
Gill-specific transcriptional responses of female adult medaka over 2-week feeding experiment for different Fe levels (27.4, 162 and 657 mg Fe/kg). Values (n = 3) are expressed as mean fold change ± SD against day 0, and significant differences (*p* < 0.05, one-way ANOVA) from day 0 (ctr) were marked with asterisk.

For the female adult fish, fish gills from different treatments were screened for Fe absorption related genes expression (Fig. 4). Among the 8 studied genes, *hep2* was not detected and the expression level of *fpn1*, *tfr*, *hep1*, *dmt1* and *fth* did not change significantly (*p* > 0.05, two-way ANOVA) over 2 weeks. For the rest two genes, all the treatments showed a variable (not significant, *p* > 0.05, two-way ANOVA) down-regulation of *tf* (i.e., 0.03–0.7 fold change) and significant (*p* < 0.05, two-way ANOVA) up-regulation of *dcytb* (i.e., 0.8–13.5 fold change) on 7–14 day.

**Figure 5:**
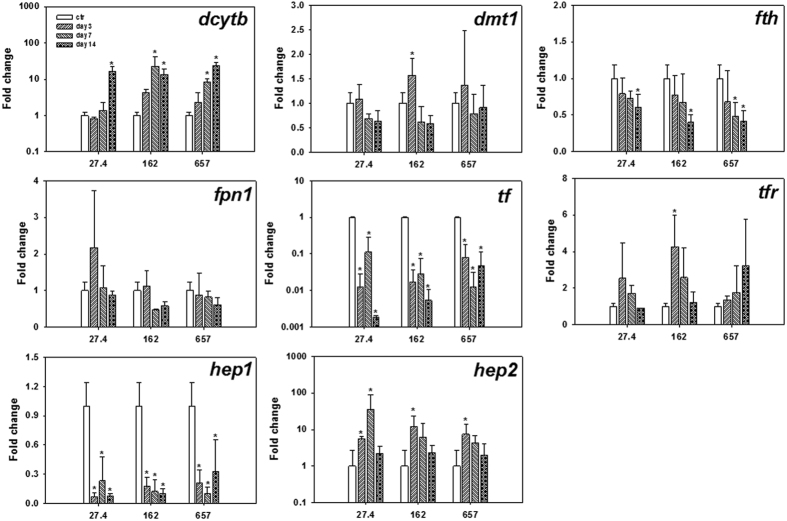
Intestine-specific transcriptional responses of female adult medaka over 2-week feeding experiment for different Fe levels (27.4, 162 and 657 mg Fe/kg). Values (n = 3) are expressed as mean fold change ± SD against day 0, and significant differences (*p* < 0.05, one-way ANOVA) from day 0 (ctr) were marked with asterisk.

Fish intestines from different treatments were screened for Fe absorption related gene expression (Fig. 5). Significant (*p* < 0.05, two-way ANOVA) down-regulation was observed for *hep1*, *tf* and *fth*. Specifically, *hep1* and *tf* were significantly (*p* < 0.05, one-way ANOVA) down-regulated in all treatments and fluctuated over 2-week exposure (i.e., 0.04–0.3 fold change for *hep1* and 0.002–0.1 fold change for *tf*), while *fth* was significantly (*p* < 0.05, one-way ANOVA) down-regulated (ca. 0.5 fold change), mostly on day 14. Up-regulation was observed for *hep2* and *dcytb*. Significant (*p* < 0.05, one-way ANOVA) up-regulation of *hep2* was only detected for 27.4 mg Fe/kg treatment on day 3 and 7, while *dcytb* was up-regulated for all the treatments from day 7 and all reached a significant (*p* < 0.05, one-way ANOVA) high level on day 14 (i.e., 13.4–23.8 fold change). The expression level of *dmt1* (i.e., 0.5–1.5 fold change), *fpn1* (i.e., 0.4–2.2 fold change) and *tfr* (i.e., 0.4–4.2 fold change) fluctuated over 2-week exposure period.

### Quantitative Fe requirement

**Figure 6:**
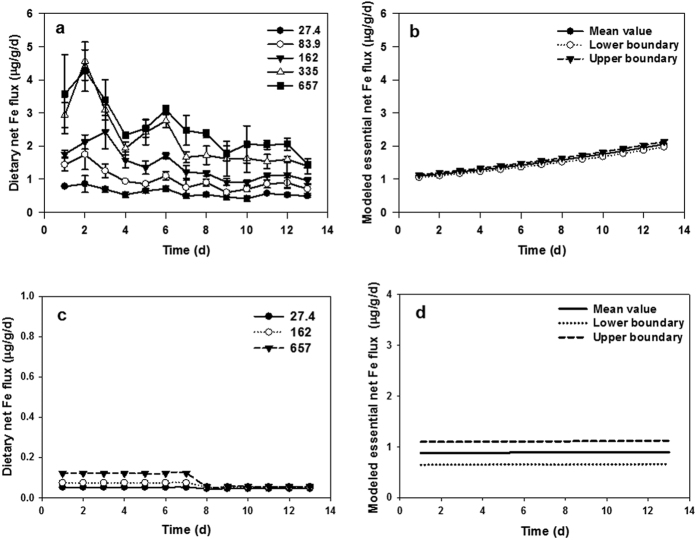
Determined dietary net Fe flux in the feeding experiment (**a**) larval fish and (**c**) female adult fish; Estimated essential net Fe flux using modified fractional model (**b**) larval fish (k_e_: 0.023 (0.017–0.029) d^−1^; g: 0.08 d^−1^) and (**d**) female adult fish (k_e_: 0.034 (0.022–0.045) d^−1^; g: 0.01 d^−1^), lower and upper boundary was defined as 95% confidential intervals of k_e_ (as shown in the bracket).

In the present study, the estimated essential net Fe flux using modified model for larval and female adult medaka was 1.09–2.16 and 0.88–0.90 μg/g/d, respectively (Fig. 6b,d). Consistently, the supplementary level of 80 mg Fe/kg feed (i.e., 0.71–1.75 μg/g/d) was determined to be sufficient for the normal development of larval medaka based on the biokenetic and transcriptional responses. However, estimation of essential net Fe flux in female adult fish from the feeding experiment was not applicable since all the fish were considered to be under sub-optimal conditions (see discussion). This modified fractional model can be further applied to other life stages of medaka as well as other fish species with known coefficient a and b (Table 3). Generally, the estimated essential net Fe flux (Table 3) varied among different fish species, ranging from 0.29–1.64 μg/g/d.

## Discussion

High mortality of marine larval fish has long become the bottleneck of marine aquaculture. Understanding their basic nutrient requirements and regulations are crucial in advancing this industry. Currently, the intestinal Fe absorption was regarded as the major route for post-larval marine fish^22^. Consistently, negligible waterborne Fe uptake (i.e., <0.097 ng/g/d) of the female adult fish was observed in the present study, when compared to the dietary assimilated Fe (ca. 0.1 μg Fe/g/d, Fig. 6). Also, intestine showed more dynamic changes than the gill among the screened Fe absorption related genes for the female adult fish (Figs 4 and 5). However, all three Fe supplementary levels (i.e., 27.4, 162 and 657 mg Fe/kg) showed similar low level of Fe flux (i.e., 0.046–0.12 μg/g/d), which was far less than the estimated 0.88–0.90 μg/g/d required by the regular kept female adult fish (Fig. 6d). Previously, the AEs of Fe (added as FeSO_4_) in coho salmon and rainbow trout were more than 10% in most cases^23^, while the female adult medaka showed poor Fe (added as FeCl_3_) AE (i.e., <2%) right after 1 week of exposure. Besides, genes involved in intracellular Fe transportation (i.e., *tf*: 0.002–0.1 fold change) and storage activities (i.e., *fth*: 0.5 fold change) in intestine were down-regulated. By further comparing the estimated daily Fe loss (i.e., 0.07 μg/g/d) in zebrafish^24^, it was therefore likely that the female adult fish could only absorb the basic life sustaining amount of Fe. In order to sustain the Fe supply, several strategies may be adopted by the female adult medaka. Since this deficiency was induced by the less bioavailable ferric ion in the present study, a significant up-regulation of the ferrireductase gene (i.e., *dcytb*) was observed on day 7 (i.e., 7.5–23.0 fold change and continued to be highly expressed on day 14 (i.e., 13.4–23.8 fold change). Previous study showed that the intestinal ferrireductase activity of rainbow trout (*Oncorhynchus mykiss*) increased significantly (*p* < 0.05) at week 4 in low Fe diet treatment (i.e., 33 mg Fe/kg feed)^25^. Another probable strategy was the down-regulation of the putative Fe regulator *hep1*, which then created a favorable condition for the Fe importation process^26^. The *hep1* was found ubiquitously expressed in different organs of fish, with the highest expression level in liver^27^,^28^. Although the *hep1* expression level was comparatively low, the intestine showed more rapid and dynamic expression change than the liver when facing bacterial challenge, presumably due to its role as the first barrier^28^. In addition, the present study suggested that the intestinal *hep1* expression could respond dynamically to the iron status. Additionally, increasing the number of transporters (i.e., *dmt1* and *fpn1*) may not be adopted by the female adult fish as an instant solution for the Fe deficiency, because no modulation of these two gene expression level was observed. However, it may be adopted in the prolonged deficiency challenge, and such up-regulation of *dmt1* and *fpn1* in gill and intestine of zebrafish (*Danio rerio*) was observed after feeding with low Fe diets (33 mg Fe/kg feed) for 10 weeks^29^. Although these proposed mechanisms may be favorable for the increase of AE in the prolonged exposure, the female adult fish appeared to be poor in assimilating less bioavailable Fe (i.e., ferric Fe) in the present study.

Comparatively, larval medaka was more efficient (i.e., >2 folds AE) in absorbing ferric Fe when fed the same level of Fe supplemented diet (Fig. 2b,d), probably due to the up-regulation of Fe importer (*dmt1*; ca. 2 folds) during this stage. Additionally, waterborne Fe uptake also contributed more (i.e., >10 folds k_u_) to the final Fe intake in the larval fish than the adult fish (Fig. 2a,b), especially when facing a dietary Fe deficiency. In the present study, although a significant (*p* < 0.05, one-way ANOVA) increase of Fe AE on day 7 (from 14.5% to 18.5%; Fig. 2b) occurred for the larval fish when fed 27.4 mg Fe/kg diet, the total dietary assimilated Fe only covered 12% of the final accumulated Fe after mass balance estimation assuming no Fe efflux. Therefore, most of the absorbed Fe was likely coming from water, as shown by the significantly (*p* < 0.05, one-way ANOVA) higher k_u_ detected on day 14. Furthermore, the larval fish (i.e., 27.4 mg Fe/kg) retained a significantly (*p* < 0.05) higher total body Fe than the other treatments (Table 2). Similar phenomenon was also observed in zebrafish after feeding on low Fe diet (33 mg Fe/kg feed) for 10 weeks^29^, indicating that dietary Fe deficiency would induce Fe hyper-accumulation from water. Indeed, Cooper and Bury found that the rainbow trout fry (*Oncorhynchus mykiss*) could potentially acquire 85% of its daily required Fe from water through gill, even at very low water Fe (both ferrous and ferric ions) concentrations (i.e., pico-molar)^30^. Differ from the freshwater fish, marine fish drink water, which makes the intestine as a waterborne Fe uptake site in addition to gill^22^. This probably provided the fish access to the large pool of ferric oxide in marine environment in addition to the dissolved Fe (i.e., 0.097 μg/L in this study). However, how different Fe agents contribute to the high accumulation of Fe by the larval fish under dietary Fe deficient conditions remains to be further examined. Transcriptionally, such low Fe diet (i.e., 27.4 mg Fe/kg feed) treatment led to an extraordinary high up-regulation of *hep2* in the larval fish, as well as in the intestine of female adult fish. Although hepcidin functions primarily as a Fe regulator in human^31^, the multiple homologies of hepcidin gene may play different roles in fish^19^. In marine medaka, the sequence of HEP2 and HEP1 peptide shared 63% similarity^27^. It was also possible that HEP2 specifically binds to FPN1, but has no inhibitory effect on the Fe exportation. In this way, the up-regulation of *hep2* can competitively decrease the inhibitory effect of HEP1, which favors the Fe internalization process. However, there is no study on this issue, and future verification is strongly needed.

The larval fish turned to control the dietary assimilated Fe once the dietary assimilated Fe met the estimated essential net Fe flux (ca. 1 μg/g/d; Fig. 6b). Biokinetically, the AE decreased in parallel with the increase of Fe supplementary level. This was probably achieved through the over expression of *hep1* at high supplementary levels (162 and 657 mg Fe/kg). However, this was not sufficient to keep a constant body Fe concentration (Table 2) at different supplementary levels (i.e., 83.9–657 mg Fe/kg), since the total assimilated Fe also increased positively with the supplementary level (Fig. 6a). An enhanced excretion route should be developed. In mammals, 12–14 μg Fe/kg/d was lost through the sloughing of gastrointestinal epithelial cells^32^. Similar strategy may also be adopted by fish^22^, but the underlying mechanism warrants future study.

Quantifying the exact Fe requirement in fish is crucial due to the essentiality of Fe to fish. Generally, higher Fe flux was observed in the early life stages (i.e., larval and juvenile) than the late stages for the investigated fish species (Table 3). However, higher daily net flux does not mean that high level of Fe should be included in the feed, primarily because the larval fish typically have a higher ingestion rate^33^. Currently, live feeds (e.g., rotifer, *Artemia* and copepods) are commonly used in larval aquaculture. The Fe concentration in these live feeds was typically 63–371 mg/kg dry weight^34^. The ingestion rate of larval fish was correlated with its body weight and estimated to be 10% of wet weight per day (Model 2; dry weight: 0.2–10 mg; temperature: 25 °C)^35^. Therefore, the daily assimilated Fe (assuming the AE of 10%) from live feeds was roughly estimated as 0.63–2.71 μg/g/d, which indicated that Fe deficiency was unlikely to occur for larval fish when fed with sufficient live feeds. Besides, some other complementary strategies (e.g., epidermal uptake) may also be adopted by the larval fish^36^. A complete understanding of these strategies would be beneficial for advancing larval nutrition study. However, Fe deficiency would most likely occur in female adult fish, especially when the added Fe source was less bioavailable. Assuming a feeding rate of 3% wet weight and a dietary Fe concentration of 150 mg/kg, an AE should be 22.2% in order to meet the daily flux of 1 μg/g/d. Therefore, increasing the bioavailability of Fe in the fish feed was critically important, since intestinal Fe uptake was the main Fe route for most of post-juvenile marine fish.

To sum up, the present study investigated the Fe regulation and requirement in two critical stages of marine medaka (i.e., larval and female adult). The larval fish reacted actively to different Fe supplement levels by increasing its waterborne Fe uptake under dietary Fe limited condition. Their dietary Fe AEs decreased in line with increasing dietary Fe supplementary levels, which may be partly achieved by the up-regulation of the iron regulation gene (*hep1*). In contrast, the female adult fish appeared to be relatively inert to different Fe supplementary levels due to the low bioavailability of added Fe source (ferric ion). Our feeding experiment suggested that 0.71–1.75 μg/g/d (i.e., 83.9 mg Fe/kg feed) was sufficient for the normal growth of larval medaka. For female adult fish, the optimal Fe net flux was 0.88–0.90 μg/g/d. The modified fractional model suggested a variable Fe flux in different fish species, while the early life stages (i.e., larval and juvenile) typically had a higher Fe flux than the late life stages.

## Methods

### Stock animals

Marine medaka (*Oryzias melastigma*) were kept in the recirculating natural seawater for several generations (>2 years) in the Coastal Marine Laboratory (Clearwater Bay, Hong Kong). Brood stock medaka were reared in separated tanks (1/2 water refreshed daily) under constant temperature (25 °C) and fed live *Artemia* nauplii twice a day (total 3% body wet wt.). Fish eggs were collected daily and incubated at 25 °C in daily refreshed seawater until hatching. Live feed rotifer was used during the larval stage (ca. 4 weeks).

### Whole life stage Fe content assay

Fish from the newly hatched larvae to the spawning adults were sampled (n = 95 in total) for their body weight and Fe content assays. The relationship between body Fe content (B_t_, ng) and weight (W_t_, mg) was established following the method of Shearer^37^:


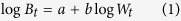


Coefficients *a* and *b* were determined for different life stages (i.e., larval, juvenile, young adult, adult female and adult male). Coefficient *b* can be used to qualify the Fe status at specific stage. When b < 1, Fe requirement was relatively low, and the fish body Fe concentration kept decreasing. When b  = 1, Fe requirement was proportional to the growth, and the fish kept a constant body Fe concentration. When b > 1, Fe requirement was relatively high, and the fish body Fe concentration kept increasing.

### Diet preparation

Semi-purified basal diet composed of casein, rice starch, fish oil, egg yolk, vitamin premix (e.g., ascorbic acid 0.92 mg/g) and mineral mix (without Fe) was prepared exactly the same as in the previous study^12^, with the assayed crude protein, crude lipid and ash to be 31.5%, 14.4% and 4.2%, respectively. Five nominal Fe supplementary (i.e., 0, 80, 160, 320 and 640 mg Fe/kg feed) diets were prepared respectively by spiking stoichiometric amounts of FeCl_3_.6H_2_O (>99%, Sigma Aldrich) stock solution (i.e., 4.3 g Fe/L). Correspondingly, the final Fe concentration in each diet was 27.4, 83.9, 162, 335 and 657 mg/kg, respectively. Feeds were stored at 4 °C until use. All the fish were acclimated to this synthetic diet (containing 162 mg Fe/kg added as FeSO_4_) for 3 days prior to the experiment.

### Gross growth parameters

Fish were starved for 24 h prior to length and weight determination. Calibrated photos of fish were taken and length was measured using Image J (National Institutes of Health, Bethesda, MD, USA). Fish were subsequently blotted dry to remove the surface retained water before wet weight measurement. Fish were then dried at 55 °C for 2 days and used for dry weight and metal concentration analysis. Considering the relatively slow growth rate (i.e., less than 1%/d) at the adult stage, body length and weight change were not considered during this short exposure period (i.e., 2 week).

A Perkin-Elmer 7000DV inductively coupled plasma optical emission spectrometry (ICP-OES; Wellesley, MA, USA) was used to determine the total body Zn, Cu and Fe concentrations. The dried samples were digested in nitric acid (70%, Aristar grade BDH, Poole, UK) at 80 °C overnight. Certificated standard material DORM-4 (National Research Council Canada, Ottawa, Canada) was used as the reference standard (agreement with the standard was within 5%) and standard Zn, Fe and Cu solutions (Perkin-Elmer, Wellesley, MA, USA) were used to quantify the metal concentrations (expressed as mg/kg, dry wt.). For the larval fish, whole body (n = 3) was included, while liver, gill, ovary and carcass (the rest) were dissected for the female adult fish (n = 3). Besides, natural seawater Fe concentration was measured using DGT-technique^38^. Briefly, the DGT device was deployed in the seawater for 3 days and the resin was retrieved for metal analysis following the manual description. The measured liable Fe concentration in the seawater was 0.097 μg/L.

### Biokinetic responses

Radiotracer technique was applied to determine the Fe AE of medaka. The radiolabelled diets were prepared similarly as in previous study using ^59^FeCl_3_ stock (specific radioactivity 3.6 μCi/mL)^12^. Additional stoichiometric amount of stable FeCl_3_ was added respectively to resemble the same Fe supplementary level used in the feeding experiment. AE assay was performed following the well-developed pulse chase feeding technique^39^ and the value was determined as the retained percentage of Fe radioactivity after 28 h depuration.

The k_u_ was determined following the method of Cooper and Bury^30^. Briefly, fish (5 individuals for larval fish and 1 individual for female adult fish) was transferred to 200 mL plastic beakers (n = 3) with 100 mL synthetic seawater^40^ for acclimation (2 days). Daily routine feeding was performed during acclimation (using exactly the same feed as the feeding experiment). Trace amount of ^59^FeCl_3_ was added in the synthetic seawater to achieve the final Fe concentration of 0.056 μg/L (radioactivity 5 μCi/L). During experiment, fish were gently transferred into the exposure medium and their radioactivities were assayed at 3 h. Besides, the radioactivity of water samples (i.e., 3 mL) was checked before and after the experiment to confirm that the variance of the concentration was within 10%. All the data were obtained with a propagated error of <10%, suggesting that the results were reliable. The k_u_ (L/g/d, based on wet wt.) was estimated using the first-order metal uptake kinetics^41^, as following:


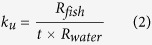


where R_fish_ (ccpm/kg) is the radioactivity in fish, t (h) is the uptake period, and R_water_ (ccpm/L) is the radioactivity in the uptake medium.

### Transcriptional responses

Larval fish (5 individuals, n = 3) were rinsed for 3 times and the surface retained water was removed using pipette. For the female adult fish, 2 individuals (n = 3) were sacrificed by shock ice chilling. Gill and intestine were sampled due to their predominant roles in Fe absorption. Samples were submerged in liquid nitrogen immediately and further stored at −80 °C until RNA extraction.

Total RNA was isolated from 10–20 mg frozen fish tissues using the PureLink® RNA Mini Kit (Ambion, cat. no. 12183555). Briefly, the frozen fish tissue was immediately mixed with 0.7 mL TRIzol reagent (Invitrogen) and homogenized using a plastic mini grinder (Biorad, cat. no. 1632146). The lysate was further homogenized using a mini homogenizer (Ambion, cat. no. 12183026) by centrifuging at 12000 *g* for 2 min at 25 °C. The filtrate was preceded for total RNA extraction by strictly following the manual of the kit. After DNA removal (Turbo DNA-free kit, Ambion, cat. no. AM1906), the RNA concentration was quantified using a NanoDrop^TM^ 3300 Fluorospectrometer (Thermo Scientific) and its integrity was checked (all RIN values were larger than 7) using an Agilent 2100 Electrophoresis Bioanalyzer Instrument (Agilent Technologies). RNA (i.e., 1 μg) was used for cDNA synthesis using iScript™ cDNA Synthesis Kit (Biorad, cat no. 170–8891). The synthesized cDNA was store at −80 °C until usage (<30 day).

The quantitative real-time PCR was conducted on a LightCycler 480 Instrument II (Roche Diagnostics GmbH) with iTaq™ SYBR® Green Supermix (Biorad, cat no. 1725851). For normalization, the 18s gene was used as reference gene^27^. Target gene sequences in marine medaka (*Oryzias melastigma*) were either obtained from existing studies in this species^27^,^42^ or identified by blasting target gene from related species (i.e., *Oryzias latipes* and *Danio rerio*) against its whole genome assembled transcripts^43^. The RT-qPCR primer pairs (Table 1) were designed with the guidelines of a production size 150–250 bp and a T_m_ of 60 °C using Primer 3.0^44^,^45^. Larval fish were assayed for all the 9 genes. Since both gill and intestine were intact organs containing blood vascular systems^46^, they were also screened for all the 9 genes.

All RT-qPCRs were performed in a 10 μL volume containing 1 μL cDNA template, 5 μL SYBR^®^ Green Supermix (with ROX), 0.2 μL of each primer (10 μM) and 3.6 μL nuclease-free water. The thermo-cycling conditions were as the following: 95 °C for 5 min, followed by 45 cycles consisting of 95 °C for 10 s and 60 °C for 45 s, and finally holding at 4 °C. A melting curve was generated for every PCR product to confirm the specificity of the reaction, and dilution series (×5) were prepared to check the efficiency of the reactions. Each sample was assayed in triplicate alongside with a negative control. Relative mRNA expression (fold change) was determined using the 2^−ΔΔCt^ method^47^.

### Quantitative Fe requirement

Dietary net Fe flux (Q_t_, μg/g/d) can be calculated as the following:


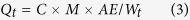


where C is the food Fe concentration (μg/g), M is the recorded weight of daily ingestion feed (mg).

The theoretical essential net Fe flux (F_t_, μg/g/d) can be estimated from the daily growth retention (G_t_, ng/d) and endogenous loss (E_t_, ng/d), as following:


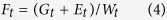


which can be further transformed as following using modified fractional models^12^.


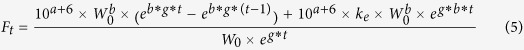


where W_0_ is the initial wet body weight (g), k_e_ is the efflux rate constant and g is the growth rate (d^−1^). Values of k_e_ and *g* for specific stages were determined followed the method of Zhang and Wang^33^. Briefly, fish of different life stages were fed radio-labeled diet for 5 days and depurated in the clean seawater for 19 days. The retained radioactivity in the fish was assayed every 2 days. k_e_ was calculated as the slope of the ln transformed percentage decline and *g* was calculated as the ln transformed weight daily percentage increase. Besides, literature available data on coefficient a, b and *g* in some fish species were gathered and the theoretical essential net Fe flux was calculated using this modified fractional model.

### Ethic statement

All the methods were carried out in accordance with the approved guidelines. In addition, all the experimental protocols were approved by the Hong Kong University of Science and Technology safety committee (Hong Kong SAR, China).

### Statistical analysis

Comparisons between different treatments for different growth parameters and k_u_ were performed using SPSS 16.0 (SPSS Inc., Chicago, IL, USA) through one-way analysis of variance (ANOVA; Turkey test). Two-way ANOVA (Turkey test) was adopted for AE and gene expression analysis, and one-way ANOVA was further used to analyze its temporal variation for a fixed supplementary level. The significance was determined at the level of 0.05.

## Additional Information

**How to cite this article**: Wang, J. and Wang, W.-X. Novel insights into iron regulation and requirement in marine medaka *Oryzias melastigma*. *Sci. Rep*. **6**, 26615; doi: 10.1038/srep26615 (2016).

## Figures and Tables

**Table 1:** Oligonucleotide primers used in this study.

Gene	Sequence 5′-3′	Accession	Similarity (%)	Annotation
*18s*	F: CAGCGTCCGGGAAACCAAAGTCTT	DQ105650.1	100	18s
	R: TGGTGGTGCCCTTCCGTCAATTCCT			
*dcytb*	F: ACTGTAGACGTGGATGGGC	XM_004081752.2	93	cytochrome b reductase 1
	R: TTGACTTCCACAACACTGCC			
*dmt1*	F: TGGCTGATGGTTGAATTGGC	XM_004069131.2	94	divalent metal ion transporter
	R: TGGTGATGAGAACTCCTGCC			
*fth*	F: CTGTCTTTGAACACCACGGG	HM137113.1	100	ferritin
	R: CATGGTTCTGAGTTTCGGCG			
*fpn1*	F: GGTGGTAGGAAGTGAGAGGG	XM_004081828.2	94	iron-regulated transporter
	R: TCTCCGTCTCTTTCGTAGCC			
*tf*	F: ATGAGGACAAGTGCAAAGCC	NM_001122912.1	90	transferrin
	R: CAGAGATTGCCATTGGAGCC			
*tfr*	F: ATCAAACTCCCACGACCCAT	XM_011489106.1	86	transferrin receptor
	R: ATGTTTGCAAGCTGGAGTGG			
*hep1*	F: CAATGACACTCCAGTTGCGG	HM590747.1	100	hepcidin 1
	R: TTTCCCTGATGTGGTTTGGC			
*hep2*	F: TCTCCAACATCTCCAACATCC	HM990658.1	100	hepcidin 2
	R: TCTCCAACATCTCCAACATCC			

**Table 2:** Growth performance and body metal concentration of larval fish after 2-week feeding experiment

Treatment	TCF^1^ (mg)	SLG^2^ (%/d)	SWG^3^ (%/d)	Body metal concentration (mg/kg, dry wt.)		
				Zn	Fe	Cu
27.4	5.39 ± 0.10^ab^	1.68 ± 0.26^a^	7.67 ± 0.65^a^	191 ± 6.18^ab^	356 ± 67.9^b^	11.7 ± 2.05^b^
83.9	5.88 ± 0.31^bc^	1.85 ± 0.19^ab^	7.75 ± 0.63^a^	184 ± 12.1^a^	127 ± 5.45^a^	7.96 ± 0.85^a^
162	5.67 ± 0.42^bc^	1.92 ± 0.04^ab^	8.39 ± 0.73^a^	177 ± 1.49^a^	130 ± 13.8^a^	7.61 ± 0.89^a^
335	6.01 ± 0.17^c^	2.31 ± 0.14^c^	8.63 ± 0.57^a^	186 ± 7.05^a^	138 ± 12.9^a^	6.62 ± 1.03^a^
657	4.92 ± 0.37^a^	2.09 ± 0.16^bc^	7.71 ± 0.54^a^	206 ± 12.2^b^	131 ± 8.12^a^	6.33 ± 1.24^a^

(Mean ± SD, n = 3, data in the same column sharing the same alphabet were statistically not significant).

^1^TCF: Total consumed feed;

^2^SLG: specific body length growth rate =  ln (final body length/initial body length)/14 day;

^3^SWG: specific body wet weight growth rate = ln (final body weight/initial body weight)/14 day.

**Table 3:** Estimation of essential net Fe flux in several fish species over a certain growth stage.

Fish species	Stages	a^1^	b^1^	W_0_ (g)	g (d^−1^)^2^	k_e_(d^−1^)^3^	Duration (d)	Modeled daily Fe flux (μg/g/d)	Reference
Rainbow trout (*Salmo gairdneri*)	Juvenile	−4.92	1.17	0.18	0.035	0.02	152	0.54–1.37	37
	Post-juvenile	−4.54	0.86	14.48	0.0097	0.02	455	0.29–0.55	
Rainbow trout (*Oncorhynchus mykiss*)	Post-juvenile	−4.31	0.93	53.50	0.027	0.02	56	1.47–1.64	48,49
Bighead Carp (*Aristichthys nobilis*)	Juvenile	−5.32	1.41	25.0	0.02	0.02	150	0.85–2.93	50,51
	Post-juvenile	−5.32	1.41	500	0.0046	0.02	150	1.62–2.15	
Marine medaka (*Oryzias melastigma*)	Larval	−3.35	1.64	0.001	0.08	0.02	14	1.09–2.16	This study
	Juvenile	−4.61	1.09	0.004	0.05	0.02	14	1.09–1.16	
	Young adult	−4.84	1.01	0.01	0.02	0.03	20	0.72–0.73	
	Female adult	−4.58	1.17	0.2	0.01	0.03	14	0.88–0.90	

^1^Coefficient a and b were obtained from the following equation Log body burden element (g) = a + b(Log fish wet weight (g));

^2^Fish growth rate g (d^−1^) of best performance treatment in feeding experiments was adopted.

^3^Mean value of efflux rate constant were adopted.
